# Supplementary material for: Preliminary Findings from the Gulf War Women’s Cohort: Reproductive and Children’s Health Outcomes among Women Veterans
Source: Int J Environ Res Public Health. 2022 Jul 11;19(14):8483. doi: 10.3390/ijerph19148483 (PMC9323962; doi:10.3390/ijerph19148483)
Supplement: Supplementary file 1 [file ijerph-19-08483-s001.zip › ijerph-1789653-supplementary.pdf]

International Journal of
Environmental Research and Public Health

MDPI

Brief Report

Preliminary Findings from the Gulf War Women's Cohort: Reproductive and Children's Health Outcomes among Women Veterans

Supplemental Material

Table S1. List of war time exposures and their prevalence in analytical subsets; Table S2: Association between having heard chemical alarms sounding and odds of adverse reproductive outcomes among women deployed to the Persian Gulf; Table S3: Association between having seen injured troops during deployment sounding and odds of adverse reproductive outcomes among women deployed to the Persian Gulf; Table S4: Association between having been in direct contact with enemy vehicles and odds of adverse reproductive outcomes among women deployed to the Persian Gulf; Table S5: Association between having taken pyridostigmine bromide pills and odds of adverse reproductive outcomes among women deployed to the Persian Gulf; Table S6: Association between having slept in an area with propane space heater and odds of adverse reproductive outcomes among women deployed to the Persian Gulf.

Table S1. List of war time exposures and their prevalence in analytical subsets.

Exposure[x]	Pregnancy Subset (N=86)	Child Subset (N=63)
Heard chemical alarms sounding	74.0%	81.4%
Saw troops who had been badly injured or killed	53.4%	57.6%
Encountered destroyed enemy vehicle	42.5%	49.2%
Used pesticide cream/liquid on the skin	54.8%	54.2%
Took PB pills	69.6%	71.2%
Slept in an area with fuel burning tent heater	50.7%	52.5%

[x] Exposed: $\geq$7 days of exposed; unexposed: no exposure reported or < 7 days of exposure.

Table S2. Association between having heard chemical alarms sounding and odds of adverse reproductive outcomes among women deployed to the Persian Gulf.

	Exposure status	OR (95% CI)
Difficultly conceiving	Exposed	2.04 (0.65, 7.22)
	Unexposed	1.00
Pregnancies ended in miscarriage/stillbirth	Exposed	1.04 (0.34, 3.19)
	Unexposed	1.00
High risk pregnancy [a]	Exposed	0.55 (0.18, 1.71)
	Unexposed	1.00
Pregnancy hypertension [a]	Exposed	0.93 (0.26, 3.58)
	Unexposed	1.00
Pre-eclampsia [a]	Exposed	0.48 (0.11, 2.04)
	Unexposed	1.00
Child born pre-term	Exposed	0.52 (0.11, 2.24)
	Unexposed	1.00
Child with any type of disability	Exposed	0.99 (0.33, 2.99)
	Unexposed	1.00

[a] Told by physician or medical provider. [b] Includes child hyperactivity disorder, frequent behavioral problems and/or other learning disabilities reported by participant. [c] Exposed: > 7 days of exposed; unexposed: no exposure reported or < 7 days of exposure.

Table S3. Association between having seen injured troops during deployment sounding and odds of adverse reproductive outcomes among women deployed to the Persian Gulf.

	Exposure status	OR (95% CI)
Difficultly conceiving	Exposed	2.75 (0.95, 8.29)
	Unexposed	1.00
Pregnancies ended in miscarriage/stillbirth	Exposed	**3.42 (1.15, 10.81)**
	Unexposed	1.00
High risk pregnancy [a]	Exposed	0.94 (0.30, 2.83)
	Unexposed	1.00
Pregnancy hypertension [a]	Exposed	1.04 (0.27, 3.71)
	Unexposed	1.00
Pre=eclampsia [a]	Exposed	1.18 (0.27, 4.97)
	Unexposed	1.00
Child born pre-term	Exposed	1.18 (0.26, 5.02)
	Unexposed	1.00
Child with any type of disability	Exposed	0.81 (0.27, 2.38)
	Unexposed	1.00

[a] Told by physician or medical provider. [b] Includes child hyperactivity disorder, frequent behavioral problems and/or other learning disabilities reported by participant. [c] Exposed: > 7 days of exposed; unexposed: no exposure reported or < 7 days of exposure.

Table S4. Association between having been in direct contact with enemy vehicles and odds of adverse reproductive outcomes among women deployed to the Persian Gulf.

	Exposure status	OR (95% CI)
Difficultly conceiving	Exposed	0.36 (0.05, 1.53)
	Unexposed	1.00
Pregnancies ended in miscarriage/stillbirth	Exposed	0.75 (0.15, 2.79)
	Unexposed	1.00
High risk pregnancy [a]	Exposed	0.58 (0.12, 2.20)
	Unexposed	1.00
Pregnancy hypertension [a]	Exposed	1.85 (0.43, 7.26)
	Unexposed	1.00
Pre-eclampsia [a]	Exposed	1.77 (0.33, 7.96)
	Unexposed	1.00
Child born pre-term	Exposed	0.29 (0.01, 1.78)
	Unexposed	1.00
Child with any type of disability	Exposed	0.85 (0.25, 2.81)
	Unexposed	1.00

[a] Told by physician or medical provider. [b] Includes child hyperactivity disorder, frequent behavioral problems and/or other learning disabilities reported by participant. [c] Exposed: > 7 days of exposed; unexposed: no exposure reported or < 7 days of exposure.

Table S5. Association between having taken pyridostigmine bromide pills and odds of adverse reproductive outcomes among women deployed to the Persian Gulf.

	Exposure status	OR (95% CI)
Difficultly conceiving	Exposed	0.94 (0.31, 2.97)
	Unexposed	1.00
Pregnancies ended in miscarriage/stillbirth	Exposed	0.80 (0.27, 2.47)
	Unexposed	1.00
High risk pregnancy [a]	Exposed	1.07 (0.34, 3.47)
	Unexposed	1.00
Pregnancy hypertension [a]	Exposed	1.22 (0.33, 4.69)
	Unexposed	1.00
Preeclampsia [a]	Exposed	1.05 (0.25, 4.72)
	Unexposed	1.00
Child born pre-term	Exposed	1.91 (0.28, 38.10)
	Unexposed	1.00
Child with any type of disability	Exposed	1.30 (0.32, 5.72)
	Unexposed	1.00

[a] Told by physician or medical provider. [b] Includes child hyperactivity disorder, frequent behavioral problems and/or other learning disabilities reported by participant. [c] Exposed: > 7 days of exposed; unexposed: no exposure reported or < 7 days of exposure.

Table S6. Association between having slept in an area with propance space heater and odds of adverse reproductive outcomes among women deployed to the Persian Gulf.

	Exposure status	OR (95% CI)
Difficultly conceiving	Exposed	1.20 (0.42, 3.49)
	Unexposed	1.00
Pregnancies ended in miscarriage/stillbirth	Exposed	1.13 (0.38, 3.34)
	Unexposed	1.00
High risk pregnancy [a]	Exposed	0.96 (0.31, 2.88)
	Unexposed	1.00
Pregnancy hypertension [a]	Exposed	**4.41 (1.06, 20.05)**
	Unexposed	1.00
Pre-eclampsia [a]	Exposed	4.45 (0.96, 31.95)
	Unexposed	1.00
Child born pre-term	Exposed	1.91 (0.45, 9.96)
	Unexposed	1.00
Child with any type of disability	Exposed	1.49 (0.51, 4.46)
	Unexposed	1.00

[a] Told by physician or medical provider. [b] Includes child hyperactivity disorder, frequent behavioral problems and/or other learning disabilities reported by participant. [c] Exposed: > 7 days of exposed; unexposed: no exposure reported or < 7 days of exposure.
